# Cross-sectional-derived determinants of satisfaction with physician-scientist training among Canadian MD/PhD graduates

**DOI:** 10.1371/journal.pone.0185218

**Published:** 2017-09-28

**Authors:** David D. W. Twa, Michael A. Skinnider, Jordan W. Squair, Christine D. Lukac, on behalf of the Canadian MD/PhD Program Investigation Group

**Affiliations:** 1 MD/PhD Training Program, Faculty of Medicine, University of British Columbia (UBC), Vancouver, British Columbia, Canada; 2 Centre for Lymphoid Cancer, BC Cancer Agency (BCCA), Vancouver, British Columbia, Canada; 3 Centre for High-Throughput Biology, UBC, Vancouver, British Columbia, Canada; 4 International Collaboration on Repair Discoveries (ICORD), Vancouver, British Columbia, Canada; 5 British Columbia Centre for Disease Control (BCCDC), Vancouver, British Columbia, Canada; 6 School of Population and Public Health, UBC, Vancouver, British Columbia, Canada; Universidade de Mogi das Cruzes, BRAZIL

## Abstract

Although MD/PhD programs require considerable commitment on behalf of students and learning institutions, they serve as an integral means of training future physician-scientists; individuals who engage in translational medicine. As attrition from these programs has longstanding effects on the community of translational medicine and comes at substantial cost to MD/PhD programs, we aimed to identify determinants that were associated with satisfaction among MD/PhD graduates, a feature that might inform on limiting program attrition. Anonymized data from a national survey of 139 Canadian MD/PhD alumni was analyzed. Factor analysis was conducted to evaluate the reliability of three questions that measured satisfaction and logistic regression was used to assess the association of outcomes with 17 independent determinants. Eighty-one percent of graduates were satisfied with MD/PhD training. Factor analysis confirmed the reliability of the questions measuring satisfaction. Determinants of self-reported satisfaction with physician-scientist training included co-authorship of more than six manuscripts during MD/PhD training. Additionally, protected research time at the place of current appointment was strongly associated with agreement that MD/PhD training had helped career progression. Demographic variables were not associated with any satisfaction indicator. Taken together, the majority of Canadian MD/PhD graduates are satisfied with their physician-scientist training. Project collaboration leading to co-authorships and protected research time were strongly associated with training satisfaction among graduates. If the value of collaboration can be realized among current and future physician-scientist trainees who are dissatisfied with their training, this might ultimately reduce program attrition.

## Introduction

Physician-scientists play an important role in translating research to clinical practice [1–7]. The training of physician-scientists in both research and clinical medicine affords them the unique position of serving as a bridge between these disciplines [8,9]. In Canada, MD/PhD programs function as a prominent, structured path for graduating physician-scientists and alumni of these programs are the focus of this study [4,5].

MD/PhD programs also represent a substantial investment of resources on behalf of both the university (11 of 16 current Canadian MD/PhD programs guarantee at least $20,000 in Canadian Dollars (CAD) in yearly research stipends and several institutions further subsidize over $10,000 CAD of the MD portion of MD/PhD tuition per year) and to the local and federal funding bodies that support MD/PhD students across Canada [4]. Furthermore, MD/PhD trainees also make considerable financial and time commitments to their training [5]. However, a sizeable number (10%) of MD/PhD trainees do not graduate from combined programs [10]. This phenomenon represents a significant and undesirable waste of resources, particularly in light of the 2016 termination of all federal support for each of the 16 Canadian medical schools that offer MD/PhD programs [8,9].

The literature suggests that decreased trainee satisfaction during training might underlie program attrition and longstanding commitments to sustained research involvement [6,7]. Therefore, we sought to identify determinants of MD/PhD graduate satisfaction during physician-scientist training. These findings may inform current MD/PhD students on what aspects of their training to focus on to derive satisfaction and identify experiences that MD/PhD programs may wish to emphasize to promote a satisfying learning experience. In turn, keeping MD/PhD trainee satisfaction at the forefront of the MD/PhD educational mission may also improve graduate success and longstanding commitment to serving as a physician-scientist [11–13].

## Materials and methods

Anonymized data (n = 139) was acquired from a survey conducted in 2015–16, of MD/PhD graduates (survey tool available within the supplement of [2]) from eight Canadian programs, including: the University of British Columbia (UBC), the University of Alberta, the University of Manitoba, the University of Western Ontario, the University of Toronto, McMaster University, Université de Sherbrooke, and McGill University. Collectively, these programs constitute the majority of Canadian MD/PhD graduates; 7/16 programs had no known graduates, while graduates from the University of Calgary were excluded from this survey due to the inability of being able to identify non-traditional MD/PhD graduates who did not simultaneously complete MD and PhD degrees within the same program. Among graduates with contact information (n = 181/186), 76.8% responded to the survey and there were no evident features among the non-responders, which would suggest response bias [2].

Respondents were asked three questions that measured satisfaction with physician-scientist training on a 1–5 Likert scale: (i) Overall, I am satisfied with the quality of my physician-scientist training, (ii) If I could revisit my choice, I would choose to attend a MD/PhD program again, and (iii) The combined MD/PhD degree has helped my career. Factor analysis was subsequently conducted to confirm a one-factor structure that measured satisfaction. Cronbach’s alpha was calculated to describe the internal consistency of the three questions that composed one factor, with a threshold of reliability set at ≥ 0.70.

Next, 17 variables were identified as potential determinants of training satisfaction. These included *demographic variables*: (i) sex, (ii) age, (iii) race (Asian, other or white); *training variables*: (i) greater than $50,000 CAD debt at graduation, (ii) completion of a master's degree prior to MD/PhD matriculation, (iii) matching to the residency of first choice location, (iv) specialty (common specialties, including internal medicine, pediatrics, neurology, and all forms of pathology compared to other non-surgical specialties as outlined by the Association of American Physicians and Surgeons), (v) completing a research fellowship, and (vi) completing a clinical fellowship; *appointment variables*: (i) appointment within Canada, and (ii) obtaining protected research time; *output during MD/PhD training*: (i) greater than six co-authorships, (ii) greater than three first authorships and (iii) obtaining Canadian Institutes of Health Research (CIHR) MD/PhD studentship funding; *output after MD/PhD training*: (i) serving as a principal investigator on a project within the last 36 months, (ii) publication of a co-authored manuscript within the last 36 months and (iii) obtaining a research grant since MD/PhD graduation (including local, provincial, national, and international foundations) (Table 1).

**Table 1:** The characteristics of surveyed Canadian MD/PhD alumni (n = 139). n is the number of survey respondents providing a response pertaining to the specified variable. IQR denotes the interquartile range.

Variables	n	Proportion (%)
**Outcomes: Indicators of Satisfaction**		
Satisfaction with physician-scientist training	136	85
Would choose to attend a MD/PhD program again	137	73
MD/PhD training helped career progression	138	85
**Demographics**		
Sex (male)	138	73
Age	129	Median = 37 (IQR = 34–41)
Race	133	
Asian		32
Other		9
White		59
**Training**		
Debt (>$50,000)	138	38
Prior master’s degree	133	22
First choice location for residency	136	90
Specialty	132	
Common		48
Other		52
Research fellowship	138	25
Clinical fellowship	136	42
**Appointment**		
Canadian appointment	135	82
Protected research time	138	56
**Output during MD/PhD training**		
≥ 7 co-authored manuscripts	136	51
≥ 4 first authorships	134	38
Held CIHR MD/PhD funding	137	72
**Output after MD/PhD training**		
PI < 36 months	138	38
Recent co-authorship	137	92
Awarded funding since MD/PhD	138	49

Finally, logistic regression was used to measure the independent association between each determinant and indicator of satisfaction once outcome variables were dichotomized into two groups. Responses 1–3 were taken as the reference and responses 4–5 indicated satisfaction [5]. Two-sided *P-*values < 0.05 were considered significant. Statistical analyses were performed in R (version 3.3.1) and RStudio (version 0.99.902). This work abides by the Declaration of Helsinki and was approved by the UBC Research Ethics Board (H15-02871).

## Results

### Descriptive analysis

White males accounted for the largest proportion (43%) of the Canadian MD/PhD graduate cohort. Twenty-two percent of MD/PhD graduates completed research or course-based master’s degrees prior to program matriculation, and 38% graduated with debt in excess of $50,000 CAD. Ninety percent of graduates achieved their first choice of location for residency and 54% completed either a postgraduate research (25%) or clinical fellowship (42%). Eighty-two percent of graduates remained in Canada for postgraduate training or a faculty or attending appointment, and 56% reported having protected research time. Almost all (92%) alumni had co-authored a published manuscript since MD/PhD graduation. During MD/PhD training, 51% of alumni published in excess of six co-authorships manuscripts, while 38% achieved at least four first authorships and 72% held CIHR MD/PhD stipend funding. Table 1 comprehensively lists the cohort characteristics.

Regarding the three indicators of satisfaction, 85% (115/136) of graduates were either satisfied or very satisfied with their physician-scientist training, 73% (100/137) would choose to attend a MD/PhD program again and 85% (117/137) agreed their MD/PhD training was helpful in career advancement. Collectively, this accounts for a weighted satisfaction score of 81%.

### Factor analysis

Likert responses to all three outcome variables were recorded by 97% (135/139) of the survey respondents. A correlation matrix for inter-variable comparison yielded coefficients exceeding 0.45 for each pair-wise comparison; values greater than the established |0.30| threshold. Uniqueness and factor loading were assessed to confirm that each outcome variable loaded onto one factor that measured graduate satisfaction with physician-scientist training. All uniqueness and factor loading values exceeded 0.20 and 0.65 respectively (Table 2). Cronbach’s alpha measured the internal consistency of the factor at 0.80, exceeding the threshold of reliability. Collectively, these findings indicate that the three outcome variables reliably measure satisfaction.

**Table 2:** Summary findings derived from factor analysis for the three outcome variables and a summary of the model features. Likert responses from 135 MD/PhD program graduates who answered all three questions were analyzed to confirm the one factor structure and quantify the reliability of measuring MD/PhD program satisfaction.

	Satisfaction with physician-scientist training	Would choose to attend a MD/PhD program again	MD/PhD training helped career progression
**Uniqueness**	0.54	0.22	0.48
**Factor loading**	0.68	0.89	0.72
	**Factor analysis model features**		
**Cronbach’s alpha**	0.80		
**Eigenvalue**	1.77		
**Proportional Variation**	0.59		

### Logistic regression

The sole significant determinant of satisfaction as measured by the indicator “would choose to attend a MD/PhD program again” was co-authorship of seven or more publications during the MD/PhD degree (OR: 2.24, 95%CI: 1.03–5.06, *P* = 0.045). A negative association was observed for debt in excess of $50,000 CAD.

Trends were observed between determinants and the indicator “satisfaction with physician-scientist training”. These included a positive association with serving as a principal investigator on a project within the past 36 months and obtaining a grant following MD/PhD graduation. A trend in decreasing odds of satisfaction was associated with having a faculty or post-graduate appointment in Canada. Additionally, a negative association for debt in excess of $50,000 CAD was also observed.

The indicator “MD/PhD training helped career progression” was significantly associated with two determinants of satisfaction including having protected research time (OR: 3.85, 95%CI: 1.46–11.49, *P* = 0.009) and co-authoring a manuscript within the past 36 months (OR: 5.73, 95%CI: 1.50–21.28, *P* = 0.008). Again, there was a trend in decreasing odds of satisfaction among graduates who maintain appointment in Canada.

Consistently across all three satisfaction indicators, demographic and training variables, including subspecialty training and both research and clinical fellowships, were not associated with graduate satisfaction. Likewise, the number of first authored manuscripts published during MD/PhD training (a potential indicator of training productivity) and obtaining CIHR MD/PhD training funding were not associated with satisfaction (Table 3).

**Table 3:** Logistic regression for outcome measures of satisfaction with physician-scientist training and independent variables. Data was derived from the surveyed Canadian MD/PhD alumni cohort (n = 139); data presented as odds ratio (95% confidence intervals), and (*) *P* < 0.05; (**) *P* < 0.01. N/A denotes independent variables that are not applicable to outcome measures.

Independent variables	Satisfaction with physician-scientist training	Would choose to attend a MD/PhD program again	MD/PhD training helped career progression
**Demographic**			
1. Sex			
*Male*	2.33 (0.87–6.10)	1.72 (0.75–3.86)	1.45 (0.51–3.84)
*Female*	Reference	Reference	Reference
2. Age	1.10 (1.00–1.23)	1.02 (0.96–1.10)	0.97 (0.90–1.04)
3. Race			
*Asian*	1.31 (0.45–4.38)	1.44 (0.61–3.64)	1.08 (0.38–3.31)
*Other*	0.91 (0.21–6.39)	0.79 (0.22–3.19)	0.90 (0.20–6.30)
*White*	Reference	Reference	Reference
**Training**			
1. Debt			
*>$50*,*000*	1.01 (0.39–2.73)	0.54 (0.25–1.17)	0.78 (0.30–2.04)
*≤$50*,*000*	Reference	Reference	Reference
2. Prior Master’s degree			
*Completed*	1.65 (0.50–7.46)	1.58 (0.61–4.61)	1.14 (0.38–4.24)
*None or incomplete*	Reference	Reference	Reference
3. Residency match location			
*First choice*	0.39 (0.02–2.13)	1.55 (0.45–4.83)	0.90 (0.13–3.67)
*≥ Second choice*	Reference	Reference	Reference
4. Specialty			
*Other*	0.97 (0.36–2.58)	1.43 (0.66–3.14)	1.11 (0.41–3.04)
*Common*	Reference	Reference	Reference
5. Research fellowship			
*Completed*	2.21 (0.69–9.92)	N/A	1.53 (0.52–5.64)
*None or incomplete*	Reference	N/A	Reference
6. Clinical fellowship			
*Completed*	1.54 (0.59–4.32)	N/A	0.93 (0.36–2.44)
*None or incomplete*	Reference	N/A	Reference
**Appointment**			
1. Location of appointment			
*Canada*	0.21 (0.01–1.08)	N/A	0.21 (0.01–1.10)
*Other*	Reference	N/A	Reference
2. Research time			
*Protected*	2.35 (0.92–6.36)	N/A	3.86 (1.46–11.49) **
*Unprotected or none*	Reference	N/A	Reference
3. Principal investigator on a project			
*≤* *36 months*	2.94 (1.01–10.70)	N/A	2.15 (0.78–6.93)
*> 36 months*	Reference	N/A	Reference
**Output during MD/PhD training**			
1. Co-authored manuscripts			
*≥* *7 papers*	N/A	2.24 (1.03–5.06) *	1.72 (0.66–4.69)
*0–6 papers*	N/A	Reference	Reference
2. First authorship			
*≥* *4 papers*	N/A	1.07 (0.49–2.42)	0.64 (0.24–1.73)
*0–3 papers*	N/A	Reference	Reference
3. MD/PhD funding support			
*CIHR*	N/A	0.51 (0.19–1.24)	1.76 (0.64–4.62)
*None or non-CIHR*	N/A	Reference	Reference
**Output after MD/PhD training**			
1. Co-authored paper			
*≤* *36 months*	1.31 (0.19–5.62)	N/A	5.73 (1.50–21.28) **
*> 36 months*	Reference	N/A	Reference
2. Grant funding success			
*Following MD/PhD*	2.73 (1.03–8.10)	N/A	0.84 (0.32–2.13)
*Unsuccessful*	Reference	N/A	Reference

## Discussion

Because of the resource commitment required for physician-scientist training, attrition rates in excess of 10% among American MD/PhD students remain a significant burden to training stakeholders [10,14–16]. Although Canadian MD/PhD and MD programs do not disclose specific rates of attrition, if we operate under the assumption that such rates of attrition are similar in Canada, this figure is particularly troublesome for Canadian MD/PhD programs, as such graduates are already underrepresented compared to equivalent American colleagues [15]. Canada graduated an average of 4.6 MD/PhD students per year from 2000–2006, while the US graduated approximately 261.9 students per year in this same timeframe, a figure that exceeds the number of Canadian graduates by over 6-fold, when normalized to the 2016 national populations of the United States (323 million) and Canada (36 million) [2,15]. Moreover, the recent federal funding landscape for MD/PhD students in Canada has waned substantially as a result of the 2016 CIHR funding decision which terminated national support of such programs; this further stretches resources available for MD/PhD trainees. In considering rates of attrition among MD/PhD students, others have suggested dissatisfaction with training as a key factor relating to attrition from MD/PhD and related programs, in addition to reduced commitment to research upon completion of postgraduate training [16–20]. As such, we investigated determinants associated with training satisfaction from anonymized data collected by the first survey of MD/PhD alumni in Canada.

We observed that independence from clinical duties, in the form of protected research time, is an important determinant of satisfaction, likely owed in part to the ability to manage research commitments effectively. Additionally, co-authorships were significantly associated with MD/PhD training satisfaction, independent of controlling for time since graduation (data not presented here). Co-authorships continued to be associated with training satisfaction beyond completion of MD/PhD training. Owed in part to the notion that these two outcomes encapsulated different time points in physician-scientist training, we believe this determinant robustly measures long-standing satisfaction of physician-scientists.

While it can be argued that the satisfaction-success corollary might explain the association between the number of co-authorships and satisfaction, as other currencies of success (first authorship publications, tenure of grant funding) were not also associated with satisfaction, we suggest that collaboration with deliverables (co-authorships) might supersede the argument that co-authorships are exclusive indicators of success. In turn, we believe that MD/PhD students should actively seek out collaborative opportunities that result in deliverables, such as co-authorships. Additionally, MD/PhD programs might preferentially weigh the number of co-authorships of applicants in the admissions process and could also consider emphasizing the importance of and/or financially subsiding student-led research projects resulting in co-authorships between MD/PhD students to efficiently promote satisfaction among the student body.

Our findings also underlie the notion that satisfaction among Canadian MD/PhD alumni is independent of age, race, and gender. We can therefore assume that MD/PhD programs are not admitting or catering to a specific demographic in a fashion that alters the perceived satisfaction among graduates. Specifically, while women are underrepresented among the MD/PhD graduate cohort and are less likely to have sustained research involvement after completing physician-scientist training, importantly, these findings are not ostensibly associated with training dissatisfaction [2]. With these findings in mind however, it is important to consider that our analysis may not comprehensively capture the intricacies of cultural and gender inequalities; several of the examined variables are affected by external forces and are not always exclusive to a survey respondent’s motivation. For example, the decision to publish a manuscript as a trainee is subject to the discretion of a supervisor and such discretion, whether intended or not, is at risk of being shaped by current cultural and gender determinants.

The principal negative association trends that we observed included graduating from MD/PhD programs with greater than $50,000 CAD debt and obtaining a clinical and/or research appointment in Canada. These findings may be attributable to the stress of debt upon graduation and duration of training, in addition to current physician-scientist funding landscape and paucity of funding opportunities available to Canadian physician-scientists. In Canada, physician-scientist salary support is not eligible for remuneration by means of CIHR operating grants and further, 2016 saw an end to the Physician-Scientist Salary Award competition offered by CIHR. As such, Canadian federal funding bodies might consider the importance of physician-scientist support, in the same way that the National Institutes of Health has expressed continued support for American physician-scientists.

The limitation of the present study is its power, as a result of our sample size, particularly when compared to analyses of analogous American MD/PhD cohorts. Our cohort size reflects both the smaller number of MD/PhD programs in Canada (16 current and nine with known graduates to-date) along with their brief existence in the physician-scientist training framework compared to American MD/PhD training programs. Furthermore, MD/PhD graduates from the University of Calgary were excluded from this analysis owing to the unique nature of their Leaders in Medicine program. Unlike all other Canadian MD/PhD programs, the University of Calgary permits students to complete PhDs sequentially to MD training and a sizeable proportion of students elect to complete only MD/MSc degrees. Additionally, due to the cross-sectional data collection method, we can only subjectively infer temporal causality between outcome measures and the significant determinants of satisfaction and further, we are not able to survey individuals who prematurely drop out from MD/PhD programs as these individuals are not disclosed by the respective universities surveyed in the initial study [2]. To further substantiate our current findings, future studies should also consider qualitative research methods, such as focus groups of MD/PhD graduates.

Taken together, our work identifies key determinants of Canadian MD/PhD graduate satisfaction. Collaboration in the form of co-authorship publications during MD/PhD and post-graduate training, in addition to protected research time, are significantly associated with increased satisfaction among graduates. In turn, if students can seek out publishable, collaborative projects that are actively supported by MD/PhD programs, and if Canadian federal funding bodies restore support for physician-scientists during and after training to facilitate tenure of protected research time, perhaps trainee satisfaction can be improved such that the rates of MD/PhD and physician-scientist training attrition are reduced.
